# Hydrodynamic Characteristics of the Sailfish (*Istiophorus platypterus*) and Swordfish (*Xiphias gladius*) in Gliding Postures at Their Cruise Speeds

**DOI:** 10.1371/journal.pone.0081323

**Published:** 2013-12-02

**Authors:** Woong Sagong, Woo-Pyung Jeon, Haecheon Choi

**Affiliations:** Department of Mechanical & Aerospace Engineering, Seoul National University, Seoul, Korea; University of Zurich, Switzerland

## Abstract

The sailfish and swordfish are known as the fastest sea animals, reaching their maximum speeds of around 100 km/h. In the present study, we investigate the hydrodynamic characteristics of these fishes in their cruise speeds of about 1 body length per second. We install a taxidermy specimen of each fish in a wind tunnel, and measure the drag on its body and boundary-layer velocity above its body surface at the Reynolds number corresponding to its cruising condition. The drag coefficients of the sailfish and swordfish based on the free-stream velocity and their wetted areas are measured to be 0.0075 and 0.0091, respectively, at their cruising conditions. These drag coefficients are very low and comparable to those of tuna and pike and smaller than those of dogfish and small-size trout. On the other hand, the long bill is one of the most distinguished features of these fishes from other fishes, and we study its role on the ability of drag modification. The drag on the fish without the bill or with an artificially-made shorter one is slightly smaller than that with the original bill, indicating that the bill itself does not contribute to any drag reduction at its cruise speed. From the velocity measurement near the body surface, we find that at the cruise speed flow separation does not occur over the whole body even without the bill, and the boundary layer flow is affected only at the anterior part of the body by the bill.

## Introduction

The sailfish (*Teleostei: Istiophoridae*) and swordfish (*Teleostei: Xiphiidae*) are large predators in the ocean, which have been known as the fastest fishes among sea animals. The sailfish and swordfish were reported to reach their maximum speeds of around 110 km/h [1]–[2], and 90 km/h [3], respectively. Therefore, it has been conjectured that the drag-reducing adaptations in both fishes might have evolved to reach such fast speeds and further to reduce the energy costs in usual swimming. Nevertheless, the hydrodynamic characteristics of the sailfish and swordfish have not been clearly understood and their drag coefficients remain still unknown, mainly because most studies about these fishes have been based on the observations of their morphological and behavioral features [4]–[12].

On the other hand, the boundary layer flow above a fish surface is one of the most important factors determining its hydrodynamic characteristics, because the friction drag and flow separation are directly affected by the boundary layer flow characteristics. Recently, flows over swimming fish have been investigated by numerical simulations [13]–[19] and experiments with digital particle image velocimetry [14], [20]–[25], but the main focus has been placed on the flow structure in the wake rather than on the boundary layer flow. A few studies [7], [26]–[28] have investigated the characteristics of the boundary layer flow over a fish or cetacean but only qualitatively except Anderson et al. [28] who measured boundary-layer velocities over the scup and dogfish (Re = 3×10^3^∼3×10^5^) using digital particle image velocimetry and showed that flow separation did not occur around those fishes.

It is generally acknowledged that the boundary layer flow over a fish is laminar at low Reynolds numbers (Re≤10^5^), turbulent at high Reynolds numbers (Re>6×10^7^), and laminar on the anterior part and turbulent on the posterior part at moderate Reynolds numbers (10^5^<Re≤6×10^7^), where the Reynolds number is defined by the swimming speed and total body length [7]. In the cases of sailfish and swordfish, it has been speculated that the boundary layer flows over both fishes are turbulent at their maximum speeds (Re≈7×10^7^), and turbulent boundary layer flows exist even at very low speeds (Re≈4×10^6^) because of laminar-to-turbulent transition generated by the bill [5], [10], [29]–[30]. However, these speculations have not been validated yet. Therefore, the measurements of the drags on the sailfish and swordfish and the boundary layer velocities above the body surfaces should be conducted to understand their hydrodynamic characteristics.

Interestingly, the skin types of the sailfish and swordfish are very different from each other. The adult sailfish has a number of V-shaped protrusions (bony scales) on its skin [8], [31], but the skin of adult swordfish is apparently smooth because the scales are deeply embedded within the dermis [8], [11]. The role of the V-shaped protrusions on the sailfish skin was investigated for the purpose of drag reduction by Sagong et al. [31]. However, they found that those protrusions do not reduce the skin friction unlike the ribbed structure found in the shark skin which reduces the skin friction by maximum 8% [32]–[34]. Although there were some speculations about the role of sailfish skin such as a complaint wall [29], dynamic damping system [4], or trap for air within its skin [6], they have not been confirmed either.

Another peculiar morphological feature from the sailfish and swordfish, hardly seen in other fast-moving fishes, is their long bill, a pronounced upper jaw much longer than a lower jaw, and thus they are called ‘billfish’. It has been reported that the billfish uses its bill to catch the prey or to defend itself from large predators [35]–[37]. On the other hand, it has been also suggested that the bill has hydrodynamic roles. One conjecture is that the form drag on the fish is reduced by the separation delay resulting from turbulence generation by the bill even at very low speeds [5], [29]. Another conjecture is that the friction drag on the main body is decreased due to large boundary layer thickness caused by the bill [7], [30], [38]. Videler [10] performed an experiment on the effect of roughness in a water channel flow and showed that a roughness, whose height is smaller than that on the bill of the swordfish, introduces laminar to turbulence transition at low speed. However, this early transition to turbulence by a roughness (conducted in a channel flow) cannot be interpreted in terms of the drag variation of the fish body by the bill owing to the geometry difference. Aleyev [7] compared the static pressure distributions along the dorsal, ventral and lateral midlines on swordfish models with and without bill, and showed that the bill prevents high pressure near the anterior part of head and shoulder, indicating possible reduction in the form drag by the bill. However, this result alone is again insufficient to determine whether or not the total drag is indeed reduced by the bill because of possible changes in the skin friction distribution.

As described above, there remain important fluid-mechanics issues associated with the sailfish and swordfish. Therefore, in our study, we investigate the hydrodynamic characteristics of these fishes from wind-tunnel experiments. First, fresh specimens of the swordfish and the sailfish were stuffed in good conditions (see *Taxidermy specimens* for the details). Then, the drag forces exerting on the whole bodies of both fishes were directly measured using a load cell at their cruise speeds. Also, the velocities in the boundary layer above the body surfaces were measured using a hot-wire anemometer, to see the development of boundary layer flow above each fish surface. The drag coefficients measured for the sailfish and swordfish were compared with each other and also with those of other kinds of fish. Next, the hydrodynamic role of the bill was investigated through the direct measurement of drag force, the surface-flow visualization, and the boundary-layer velocity measurement by varying the shape of bill.

## Materials and Methods

### Taxidermy specimens

**Figure 1:**
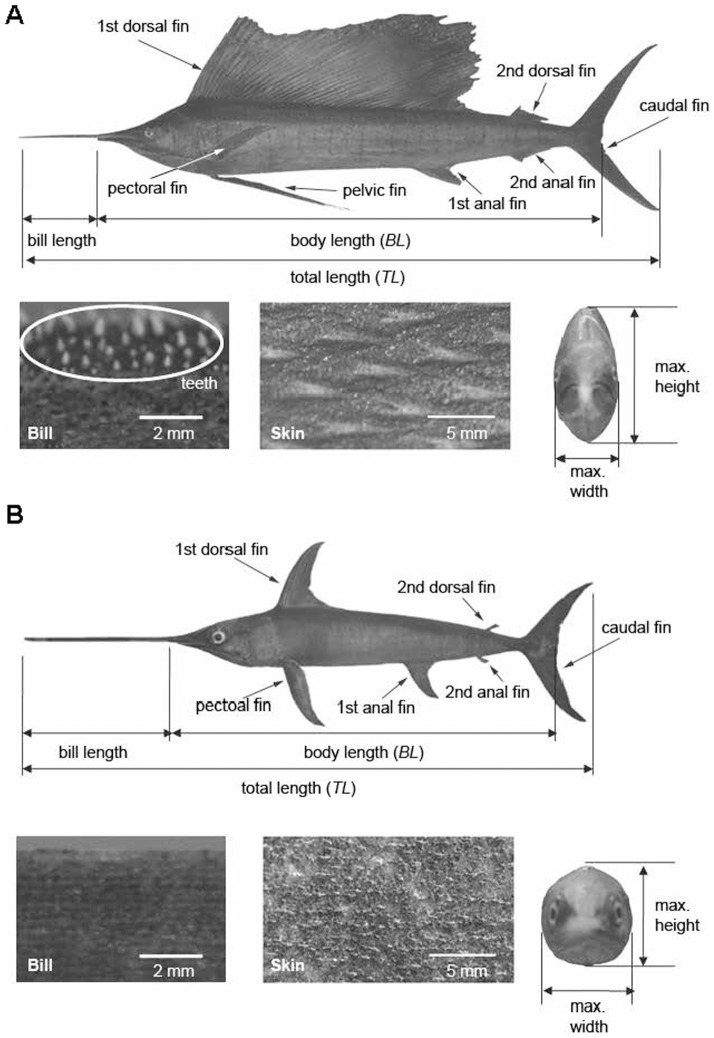
Taxidermy specimens used in the present study. In both **A** sailfish and **B** swordfish, the overall shape (top), enlarged views of bill (bottom and left) and skin (bottom and center), and frontal view of body (bottom and right) are shown.

The sailfish, *Istiophorus platypterus*, [total length (*TL*) of 2.25 m] and swordfish, *Xiphias gladius*, (*TL* of 2.0 m) were captured at the South China Sea and the Pacific Ocean, respectively. Immediately after capture, the fishes were euthanized through oxygen deprivation (de-watering), which is one of the methods in the “Guidelines for the use of fishes in research” by the American Fisheries Society, American Institute of Fisheries and Research Biologists, and American Society of Ichthyologists and Herpetologists (Bethesda, MD, 2002; http://www.nature.nps.gov/biology/iacuc/assets/docs/Module04.pdf). After they were euthanized, the factors determining the body geometry, e.g. lengths of bill, head, trunk and fins, depths, widths and girths of the trunk at several streamwise locations, inter-orbital width, etc. were measured. The sailfish was stored in ice and stuffed within 24 hours after it was delivered. The swordfish was frozen immediately after capture. To maintain the geometries of body and fins, urethane foam was placed inside the body and the fins were fully spread and fixed. The stuffing method is described in Korean Patent [39] and was also used in our recent work of Park and Choi [40]. We bought both stuffed fishes operated by the Korean Research Center of Maritime Animals. As shown later (see *Morphometrics* and Table 1), the morphometric parameters of the present taxidermy specimens are quite similar to those of previous studies [5], [7]. The shapes of the sailfish and swordfish are similar to each other, because they commonly have a long bill in front of head and a lunate caudal fin (Figure 1). Differences also exist between two fishes. For example, the sailfish has a laterally-compressed body, large sail-like dorsal fin and teeth on the jaw (Figure 1A), whereas the swordfish has a cylindrical body, a short-based dorsal fin and no teeth on the jaw (Figure 1B). The taxidermy specimens of the sailfish and the swordfish were configured with the body stretched straight with all the fins spread as shown in Figure 1. Fin grooves are developed on the body to fold down the first dorsal, first anal and pelvic fins for the sailfish, but not in the swordfish [8]. The sailfish spreads out those fins to enhance the maneuverability or stability but usually depresses them into the fin grooves during active swimming, while all the fins lie away from the body and do not rest on the body during movement in case of the swordfish [6]. Therefore, in the present study, the sailfish without first dorsal, first anal and pelvic fins, and the swordfish with all fins are considered as the standard shapes corresponding to typical swimming postures.

**Table 1:** Morphometric parameters of the sailfish and swordfish (see also Figure 1).

	Present study		Aleyev [7]		Ovchinnikov [5]	
	sailfish	Swordfish	sailfish	swordfish	sailfish	swordfish
Total length, *TL* (m)	2.25	2.00	1.83	1.85	-	-
Body length, *BL* (m)	1.74	1.28	-	-	1.50	1.65
Bill length (m)	0.29	0.56	-	-	0.25	0.56
Bill length/Body length	0.17	0.44	0.14∼0.30	0.40∼0.45	0.17	0.34
Maximum width (m)	0.12	0.19	-	-	-	-
Maximum height (m)	0.28	0.21	-	-	-	-
Maximum thickness (m)	0.18	0.20	-	-	-	-
Streamwise position of maximum thickness*	0.24	0.32	0.22	0.31	0.24	0.29
Fineness ratio**	9.67	6.40	10	5.88	-	-
Wetted area of fish (standard) *** (m^2^)	0.812	0.761	-	-	-	-
Wetted area of fish (without fins) **** (m^2^)	0.765	0.661	-	-	-	-
Wetted area of bill (m^2^)	0.0093	0.0474	-	-	-	-
Maximum cross-sectional area (m^2^)	0.0250	0.0315	-	-	-	-

*The streamwise position of maximum thickness is defined as the distance from the tip of lower jaw to the location of maximum thickness, normalized by the body length.

**The fineness ratio is defined as the ratio of body length to the maximum thickness.

***The wetted area of fish (standard) contains those of the bill, body (trunk) and all the fins for the swordfish, but the first dorsal, first anal and pelvic fins are excluded for the sailfish.

****The wetted area of fish (without fins) means those of the bill, body (trunk) and caudal fin only.

The bill of the sailfish is rounded and its length is about 17% of body length (*BL*), whereas that of swordfish is flat sword-like and its length is about 44% of body length (see Table 1). The height of protrusions (teeth) on the bill of the sailfish is about 0.5 mm, but the bill of the swordfish does not have such a distinct tooth (Figure 1). Both of the bills are covered with craters and bumps (observable through a microscope only) but their sizes are much smaller than that of teeth of the sailfish. These morphological features of the bills agree well with the observations made by previous studies [7]–[8], [10]. To investigate the hydrodynamic role of bill, experiments are performed by varying the bill shape, i.e. its length and roughness. The bill is cut out of the specimen at the tip of lower jaw, and the original and artificial bills are attached or detached at this location. In the case of sailfish, four artificial bills with different lengths and roughness heights are tested; short (2.3% of *BL*) and long (39% of *BL*) artificial bills having the same protrusion height (roughness height  = 0.5 mm) as that of the original one (17% of *BL*), and smooth (no roughness) and rough (2 mm) artificial bills having the same length (17% of *BL*) as that of the original one (0.5 mm). The roughness is placed only on the left and right sides of these bills as the teeth of original one. On the other hand, only a smooth short bill (4.3% of *BL*) is considered for the case of swordfish.

### Drag measurement

The drag forces on the fishes are measured in a closed-type wind tunnel (Göttingen type). The length of test section is 4 m (*x*) and the cross-sectional area is 0.9 m (*y*)×0.9 m (*z*). Here, *x*, *y* and *z* denote the streamwise, vertical and spanwise directions, respectively. The blockage ratios of the sailfish and swordfish in this wind tunnel are about 3% and 4%, respectively, which satisfy the criterion (7.5%) to avoid the disturbances from the wind-tunnel wall [41]–[42]. The cruising speeds of the sailfish and swordfish have been reported to be approximately 1 m/s [9], [12] and that of scombroid fish is about 1–2 *BL*/s [43]. To match their Reynolds numbers at the cruising conditions, the free-stream velocities (*u_∞_*) are taken to be from 10 m/s to 30 m/s in the present study; the Reynolds numbers based on the total length (*Re_TL_*  =  *u_∞_ TL/ν*) are from 1.5×10^6^ to 4.5×10^6^ for the sailfish, and from 1.33×10^6^ to 4.0×10^6^ for the swordfish. Here, *TL* and *BL* are the total and body lengths of each fish, respectively (Figure 1), and *ν* is the kinematic viscosity. The uniformity of mean streamwise velocity and turbulence intensity are both within 0.3% at *u_∞_* = 20 m/s.

**Figure 2:**
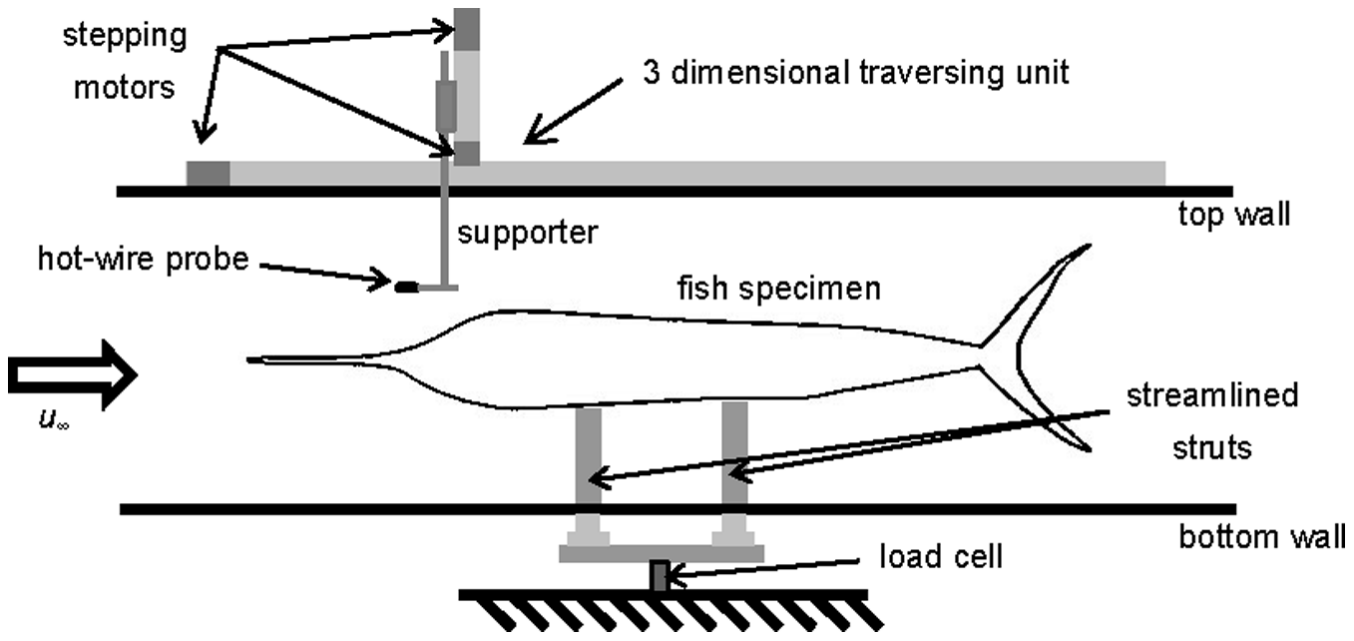
Schematic diagram of the experimental setup.

Each taxidermy specimen is located at the center of the test section by the streamlined struts and the drag force is measured with 1-axis load cell (BCL 3L, CAS, Korea) installed underneath the bottom wall of test section, connected to the specimen through the streamlined struts (Figure 2). The voltages from the load cell are amplified by signal conditioning amplifier (2310B, Vishay Micro-Measurements, USA), digitized by A/D converter (PXI-6259, National Instruments Co., USA) and then, after they reach a steady state, sampled during 30 seconds at the rate of 16 kHz to obtain a converged mean value. The drag force on each fish is determined by subtracting the drag on the streamlined struts from the total drag. The repeatability error in the force measurement is within ±1.5%. For example, the drag force on the sailfish is 1.51±0.023 N at *u_∞_* = 20 m/s. For the verification of the present force-measurement method, we measure the drag on the flat-faced circular cylinder whose length is 2 m and ratio of the length to the diameter is 8, comparable to those of fishes. The drag coefficient based on the frontal area is about 1.01 at *Re_d_* = 1.67×10^5^ which is almost same as that previously reported (0.99; [44], [45]), confirming the accuracy of the present drag measurement.

In the present study, the drag is measured on a fish stretched straight without any undulatory motion. One may argue that during active swimming the drag tends to be higher than the present one since it involves more undulatory motion [15], [16], [28], [46]–[49]. However, there has been no study on the swimming kinematics of the swordfish and billfishes [50], except that they are categorized as carangiform swimmers [51], and we do not have any information how much undulatory motion is necessary for them to generate thrust. Owing to the lack of information on their swimming kinematics, we conduct the drag measurement under the present swimming postures. Thus, the present study may provide the first measurements of drag on these fishes at coasting or gliding.

### Tuft flow visualization

To observe surface-flow patterns on the body of fish, a number of tufts are attached to one side of the body. Initially, their free ends are directed downward due to the gravitational force but change their directions with flow according to the surface-flow pattern. The tufts would vibrate severely back and forth at the location of flow separation. Thus, the separation point on the body of fish can be easily observable using this tuft visualization method.

### Velocity measurement

The velocities are measured using an in-house hot-wire anemometer and I-type hot-wire probes (55P15, DANTEC Dynamics, Denmark). The sensor used in the present study is a platinum-10% rhodium wire with a diameter of 2.5 µm and the cut-off frequency of sensor is about 25 kHz at the overheat ratio of 1.2. The output voltages from the anemometer are digitized by A/D converter (PXI-6259, National Instruments Co., USA) and sampled for 16 seconds at the rate of 16 kHz to obtain mean and rms (root-mean-square) velocities. The voltages are calibrated at the free stream with a standard 2-hole Pitot tube and a digital manometer (220DD-00100B2B, MKS Instruments, Inc., USA). A polynomial of fourth order is used as a calibration curve. To measure the boundary layer velocities along the fish body, a hot-wire probe is positioned by a three-dimensional traversing unit (resolution: 0.01 mm) controlled automatically using a computer and stepping motors (PK569-NA, ORIENTAL MOTOR Co., Japan) as shown in Figure 2. The boundary layer velocities are measured at *u_∞_* = 10 and 20 m/s. For the validation of the present velocity measurement, the mean and rms velocities in a fully turbulent boundary layer are measured and compared with those of previous studies [52]–[53], showing excellent agreements (not shown in this paper).

## Results and Discussion

### Morphometrics

Table 1 shows the morphometric parameters of the sailfish and swordfish considered in the present study. All the lengths are measured with a tape measure. The total length is measured from the tip of bill to the end of caudal fin, the body length from the tip of lower jaw to the posterior margin of the middle part of caudal fin, and the bill length from the tip of bill to the tip of lower jaw, respectively (see Figure 1). The maximum height and width are also measured. The girths are measured at intervals of 2 cm along the streamwise direction to calculate the wetted area of the body. The cross-sectional area of the body and the wetted area of fins are measured from scaled photographs. The maximum thickness is calculated as the diameter of circle having the same area as the maximum cross-sectional area. Most of the parameters in Table 1 are similar to those of previous studies [5], [7].

As shown in Table 1, the total lengths of both fishes considered in the present study are comparable to each other. The swordfish has a much longer bill whose length is about 44% of the body length, while the bill length is 17% of the body length in the case of sailfish. The ratios of the wetted area of bill to that of fish without fins are 1.2% and 7.2% for the sailfish and swordfish, respectively. The width of sailfish is much smaller than its height due to the laterally-compressed body shape, whereas both of the width and height of swordfish are similar to each other due to its circular cross-sectional body shape (see Figure 1). The fineness ratio of the sailfish is larger and its maximum thickness locates more anteriorly than those of the swordfish.

### Drag coefficients of the sailfish and swordfish

**Figure 3:**
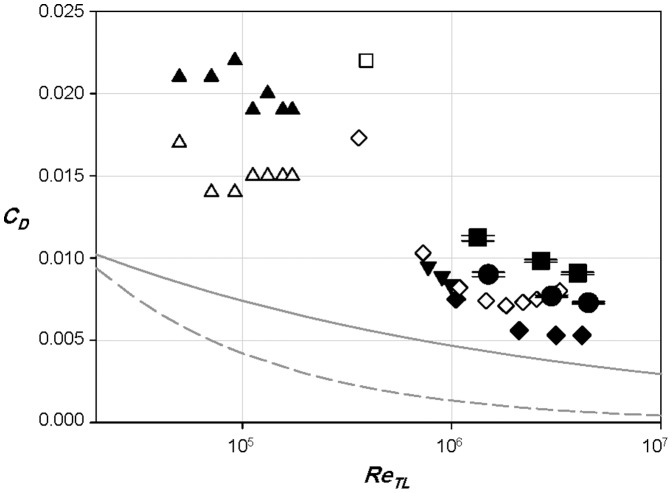
Drag coefficients of underwater animals. •, sailfish (present); ▪, swordfish (present); ▾, bluefin tuna [50]; ▴, small-size rainbow trout [26]; △, small-size rainbow trout (without fins) [26]; ♦size rainbow trout [26];⋄, pike [26]; □, dogfish [26]. The drag coefficients of all the fishes shown in this figure were measured in their gliding postures. The solid and dashed lines represent the drag coefficients of smooth flat plate in turbulent and laminar boundary layer flows, respectively. Tiny horizontal bars on the data of sailfish and swordfish denote the error bars.

The drags on the specimens of sailfish and swordfish in gliding postures, whose bodies are stretched straight, are directly measured in the wind tunnel. The Reynolds numbers correspond to their cruising conditions where the swimming speeds are about 1 body length (*BL*) per second [9], [12], [43]. In the present study, the angles of attack of all the fins are set to be nearly zero to minimize possible increases in the drag due to the fins. Figure 3 shows the drag coefficients of the sailfish and swordfish, together with those of other underwater animals such as the bluefin tuna, rainbow trout, dogfish and pike [29], [54], where the drag coefficient (*C_D_*) is based on the wetted area of the fish. All the data of the previous studies were obtained from the drag measurements by installing a fish model in a wind tunnel or towing a dead fish or fish model in a water tank, similar to the present drag-measurement method. As shown in Figure 3, the drag coefficients of the sailfish and swordfish are smaller than those of the dogfish and small-size trout, slightly larger than those of large-size trout, and comparable to those of tuna and pike. Barrett et al. [54] obtained the drag coefficient of bluefin tuna, known as one of the fast fishes, by towing a smooth rigid model without tail in a water tank. Due to the simplified shape of the fish model, the drag coefficient in their study might be underestimated. On the other hand, Webb [29] calculated the drag coefficients of pike, dogfish, and large-size trout using the drag values from the previous studies [55]–[57], but the wetted areas of those fishes were estimated to be 0.4×(total length)^2^. However, the ratio of wetted area to the square of total length varies depending on the fish. For example, it is about 0.2 for present sailfish, 0.25 for dogfish [58], 0.28 for pike [59], 0.3 for present swordfish, and 0.41 (or 0.54) for trout [59], [60], respectively. Here, the bill is excluded in the calculation of the ratios of the present sailfish and swordfish, and Musick et al. [58] and Tytell [60] used the standard length (from the snout to the end of the vertebra) and the body length, respectively, instead of the total length of fish. Therefore, owing to the uncertainty of the wetted area and different experimental conditions, it is difficult to make a firm conclusion on which fish has lower drag coefficient than others. Nevertheless, we may stress from Figure 3 that both the sailfish and swordfish have quite low drag coefficients as compared to those of many of other fishes, and the sailfish has a lower drag coefficient than the swordfish.

**Figure 4:**
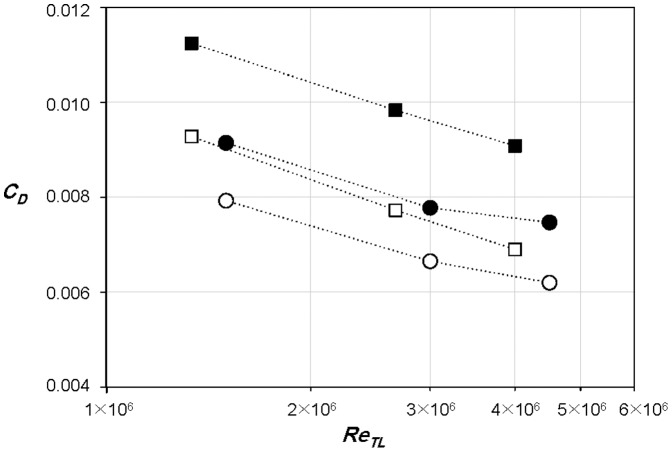
Drag coefficients of the sailfish and swordfish. •, sailfish; ▪, swordfish; ○, sailfish without fins; □, swordfish without fins.

In addition to the drag forces of the sailfish and swordfish at typical swimming postures (see *Taxidermy specimens*), their drags without median and paired fins are also measured. In the case of sailfish, all of the fins are depressed or attached to the body when it moves fast to catch a prey. As shown in Figure 4, the drag coefficient of the sailfish at a typical swimming posture is about 18% larger than that without fins, because in the presence of fins the drag increases by about 25% but the wetted area increases by only 6%. Since the fins generate the form drag and the interference drag between the body and fins as well as the friction drag, it is no wonder that total drag increases more rapidly than the increment of the wetted area. The main contributor to the drag increase is the pectoral fins which increase the drag by about 21.5%. The drag coefficient of the swordfish at a typical swimming posture is larger by about 32% than that without fins (Figure 4). The fins increase actual drag forces by about 51%, while increasing the wetted area by about 15%. In the case of swordfish, the pectoral fins and the first dorsal fin increase the drag force by 26% and 20%, respectively. The main role of the pectoral fins is known as the generation of lift force to maintain the vertical position of fish [43], [61]. Therefore, the pectoral fins increase the lift at the expense of the drag. As mentioned before, the angle of attack of the pectoral fins is set to be nearly zero, so in swimming the drag should be even larger because the attack angles are non-zero for lift generation. A similar result is also found in Webb [29] that the rainbow trout with paired fins has 20∼60% larger drag than that without fins (Figure 3).

### Velocity profiles above the sailfish and swordfish bodies

**Figure 5:**
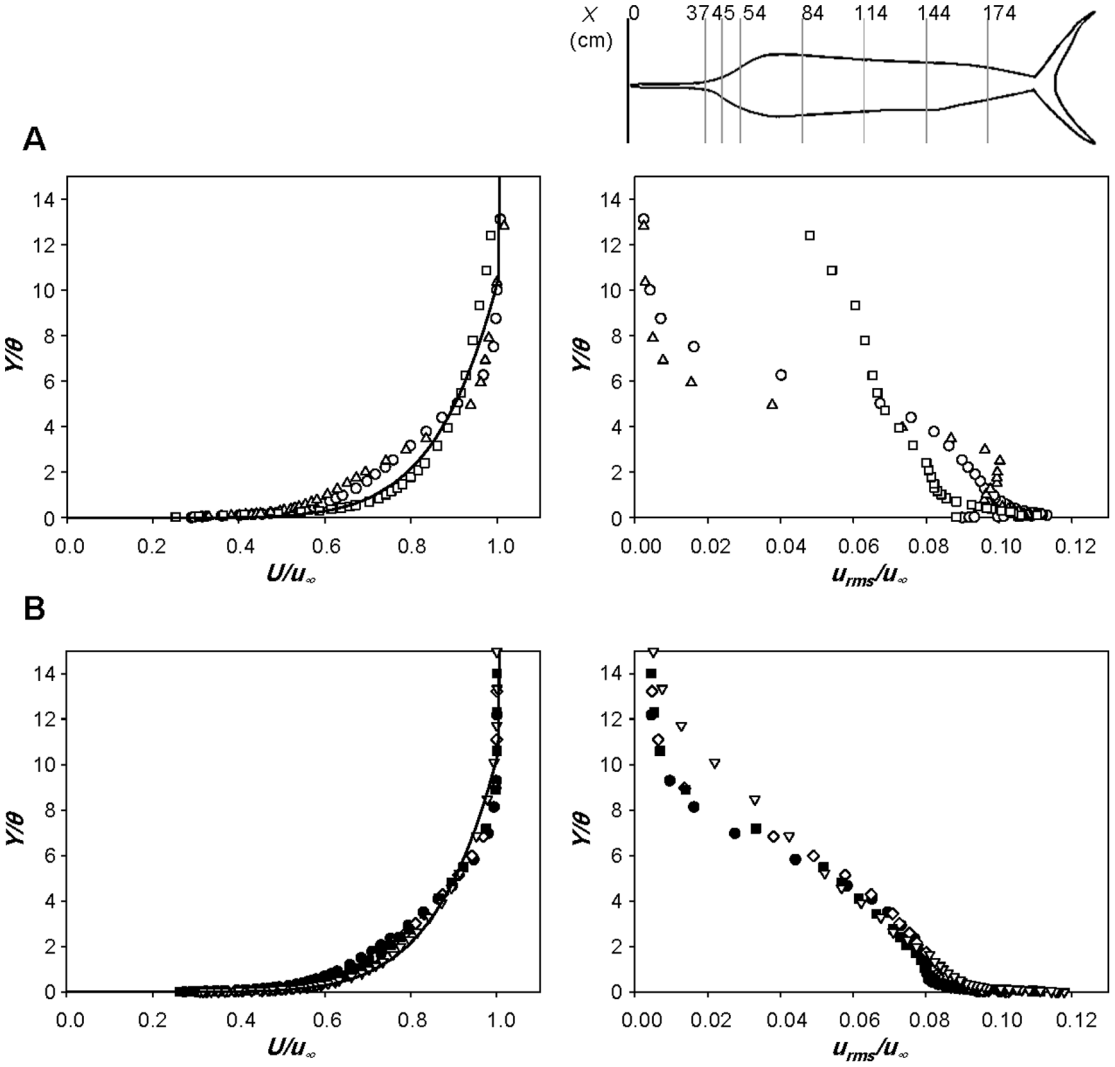
Velocity profiles over the body surface of the sailfish. Profiles of the mean streamwise velocity (left) and rms streamwise velocity fluctuations (right) at the anterior **A**, middle and posterior **B** parts of the sailfish: ○, *X/TL*  = 0.164 (37 cm; *Re_X_*  = 4.93×10^5^); △, 0.200 (45 cm; *Re_X_*  = 6.00×10^5^); □, 0.240 (54 cm; *Re_X_*  = 7.20×10^5^); •, 0.373 (84 cm; *Re_X_*  = 1.12×10^6^); ⋄, 0.507 (114 cm; *Re_X_*  = 1.52×10^6^); ▪, 0.640 (144 cm; *Re_X_*  = 1.92×10^6^); ▽, 0.773 (174 cm; *Re_X_*  = 2.32×10^6^). –, turbulent boundary layer profile (1/7^th^ power law) [45]. Here, *X* is the streamwise distance from the tip of bill, *Y* is the wall-normal distance from the body surface and *θ* is the momentum thickness (Table 2). The velocities are measured along the centerline of side surface of the sailfish at *Re_TL_*  = 3.0×10^6^. Shown at the top are the measurement locations in the streamwise direction.

Figure 5 shows the profiles of the mean streamwise velocity and rms streamwise velocity fluctuations along the centerline of side surface of the sailfish at *Re_TL_* = 3.0×10^6^. Here, *X* is the streamwise distance from the tip of bill and *Y* is the wall-normal distance from the body surface. At each streamwise location, the momentum thickness (*θ*) is calculated and given in Table 2. At all the measurement locations, flow separation is not observed and the rms streamwise velocity fluctuations near the body surface are high, indicating that turbulent boundary layer flow is maintained over the entire surface of sailfish. The local Reynolds number shortly after the tip of lower jaw (*X/TL* = 0.164) is *Re_X_* = 4.93×10^5^, which is already bigger than the critical Reynolds number for laminar-to-turbulent transition considering the roughness on the bill [62]. The velocity profiles in the anterior part (Figure 5A) change significantly along the streamwise direction. Owing to the body curvature in the anterior part, adverse and favorable pressure gradients are formed and the mean velocity is first decelerated and then accelerated at *X/TL*  = 0.164–0.200 and 0.200–0.240, respectively. Likewise, the rms velocity fluctuations are first increased and then decreased, respectively, in the anterior part of the body. In the middle and posterior parts of the body (Figure 5B), the boundary layer grows under a weak adverse pressure gradient (Table 2); the momentum thickness in this region grows like 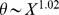
(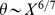 for zero pressure gradient boundary layer [45]). We also measured the boundary layer velocity at *Re_TL_*  = 1.5×10^6^, showing similar behaviors to those shown in Figure 5.

**Table 2:** Streamwise variations of the momentum thickness.

sailfish	*X* (cm)	37	45	54	84	99	114	144	159	174
	*X/TL*	0.164	0.200	0.240	0.373	0.440	0.507	0.640	0.707	0.773
	*θ* (mm)	1.606	2.035	0.649	1.731	1.912	2.352	2.935	3.243	3.080
swordfish	*X* (cm)	65	76	86	102	118	146	161		
	*X/TL*	0.325	0.380	0.430	0.510	0.590	0.730	0.805		
	*θ* (mm)	1.008	0.883	1.146	1.442	2.022	3.278	3.555		

**Figure 6:**
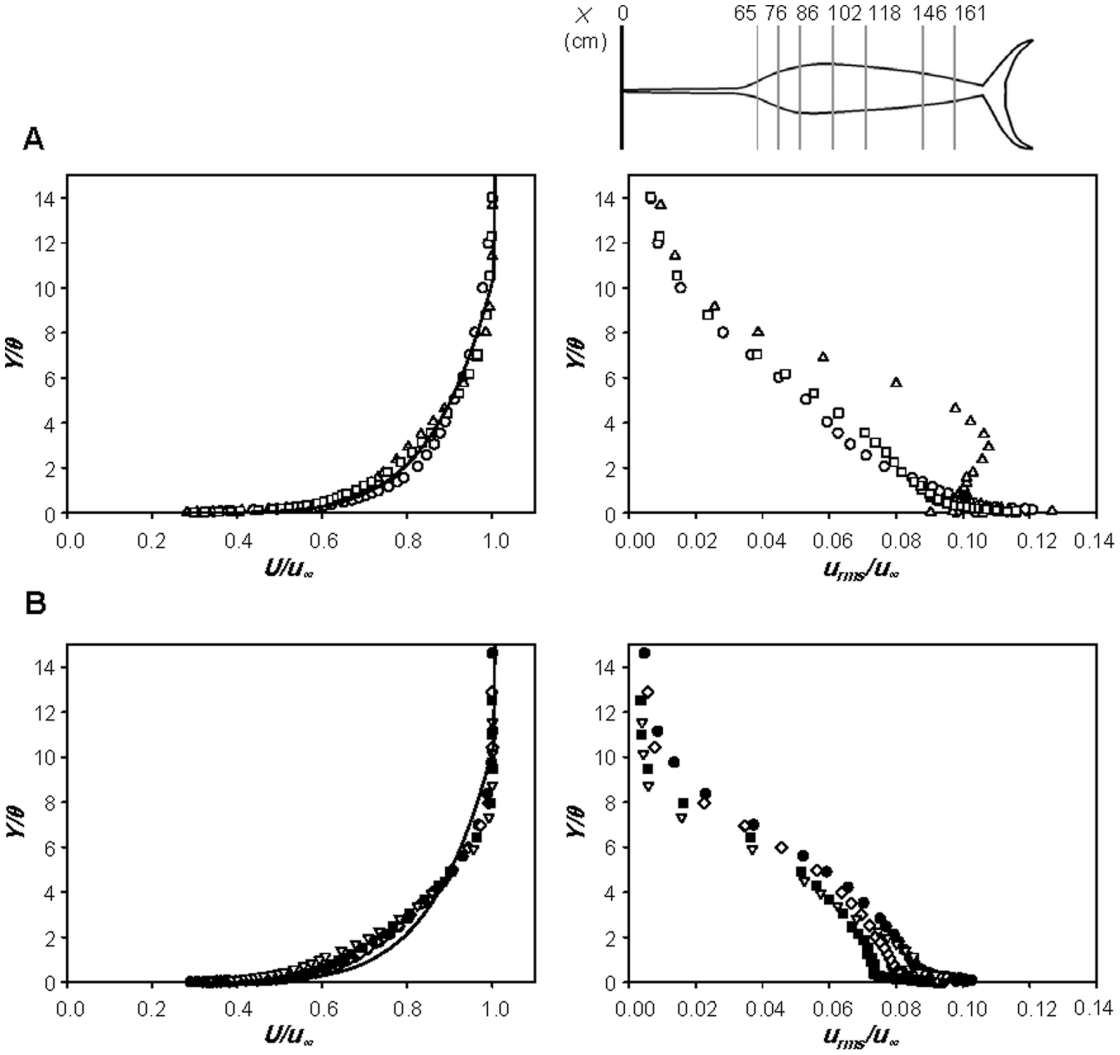
Velocity profiles over the body surface of the swordfish. Profiles of the mean streamwise velocity (left) and rms streamwise velocity fluctuations (right) at the anterior **A**, middle and posterior **B** parts of the swordfish: ○, *X/TL*  = 0.325 (65 cm; *Re_X_*  = 8.67×10^5^); △, 0.380 (76 cm; *Re_X_*  = 1.01×10^6^); □, 0.430 (86 cm; *Re_X_*  = 1.15×10^6^); •, 0.510 (102 cm; *Re_X_*  = 1.36×10^6^); ⋄, 0.590 (118 cm; *Re_X_*  = 1.57×10^6^); ▪, 0.730 (146 cm; *Re_X_*  = 1.95×10^6^); ▽, 0.805 (161 cm; *Re_X_*  = 2.15×10^6^). –, turbulent boundary layer profile (1/7^th^ power law) [45]. Here, *X* is the streamwise distance from the tip of bill, *Y* is the wall-normal distance from the body surface and *θ* is the momentum thickness (Table 2). The velocities are measured along the centerline of side surface of the swordfish at *Re_TL_*  = 2.67×10^6^. Shown at the top are the measurement locations in the streamwise direction.

The profiles of the mean streamwise velocity and rms streamwise velocity fluctuations along the centerline of side surface of the swordfish at *Re_TL_*  = 2.67×10^6^ are shown in Figure 6. Again, turbulent boundary layer flow exists over the whole surface of swordfish and flow separation does not occur. Like the sailfish, the rms velocity fluctuations are first increased and then decreased in the anterior part of the body. In the middle and posterior parts, the boundary layer velocity follows the adverse pressure gradient boundary layer characteristics, 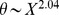
(see Table 2).

The boundary layer flow characteristics explain the reason why the position of maximum thickness is located more anteriorly in the cases of sailfish and swordfish as compared to the other kinds of fish such as the tuna. As shown in the velocity measurement data, turbulent boundary layer flows exist on the entire parts of the sailfish and swordfish, even including the anterior parts of heads. Once the turbulent velocity profile is generated at the anterior part of the fish body, early position of maximum body thickness should be preferable in terms of reducing total drag than its position on the middle part of the body, because a longer body section with decreasing body thickness in the streamwise direction has smaller skin friction there and a fuller turbulent velocity profile formed at the anterior part maintains attached flow there. On the other hand, the tuna, one of the most highly evolved fishes, possesses the thickest portion at 0.4∼0.5 of body length from the snout to maintain laminar boundary layer flow as much as possible for low skin friction [4], [26], [40], [57].

### Role of skin protrusions

The V-shaped protrusions on the sailfish skin did not reduce the skin friction in a turbulent boundary layer but each of them produced a pair of streamwise vortices that might be related to a delay of turbulent separation [31]. To investigate if these V-shaped protrusions reduce the overall drag on the sailfish, the drags on the sailfish with and without them are separately measured. Here, all of the median and paired fins are excluded to isolate the effect of V-shaped protrusions on the drag, and a smooth skin without protrusions is realized by covering the surface of fish with a very thin tape. As a result, the smooth skin reduces the drag by 5∼7%. On the other hand, the protrusions observed on the swordfish skin are tiny and much smaller than those on the sailfish skin (see Figure 1). They are the tips of the scales whose major parts are immersed in the dermis [8], [11]. The drag on the swordfish with smooth skin is decreased by 1∼4% than that of the original swordfish. From the present study, it is concluded that the protrusions observed on the sailfish are irrelevant to the capability of drag reduction because turbulent boundary layer flow is maintained over the whole body and no flow separation occurs even without the protrusions. The V-shaped protrusions increase the skin friction, acting just as a kind of roughness, in a turbulent boundary layer. However, it should be noted that the present measurements are conducted at the cruse speeds. So, flow separation may occur at their maximum speeds or due to unsteady movements such as accelerations and turning, and then the protrusions on the skin may make a role of separation delay, which we cannot confirm at this moment due to the limitation of our experimental facility.

The main function of the scales in most teleost fishes is the external protection [63]. However, due to the sparse distribution or lack of scales, the ones in the sailfish and swordfish may not play the protective role unlike those in most teleost fishes. A possible role of V-shaped protrusions on the sailfish is to aid in deposition of slime or air near the surface, which enables to reduce the skin friction [30]. However, according to Vogel [64], slime secretion is too expensive to use except in an emergency. Therefore, the role of V-shaped protrusions on the sailfish skin is not clear at this moment. The lack of scales on the swordfish may be regarded as an adaptation for reducing the skin friction.

### Role of bill

**Figure 7:**
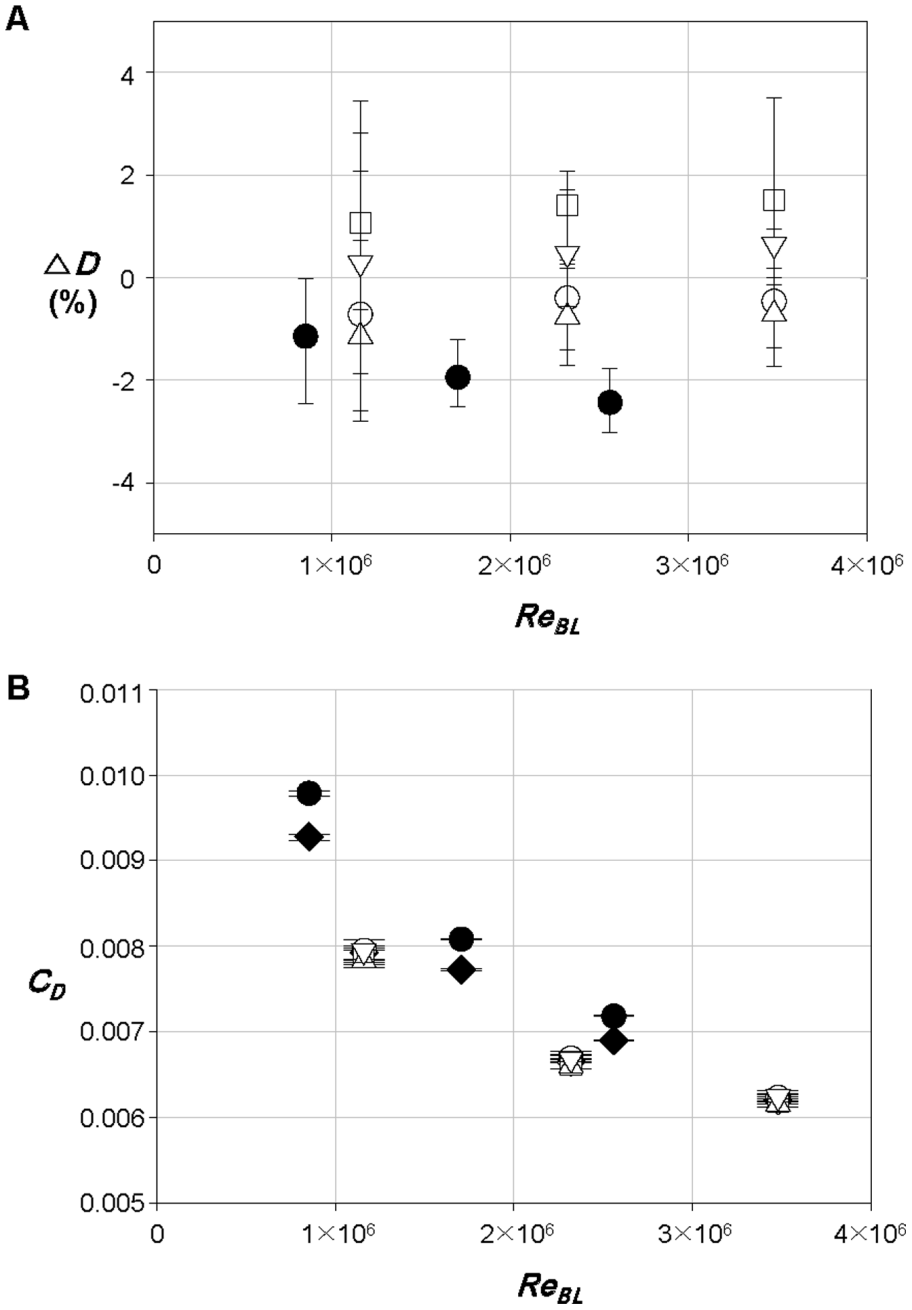
Variations of the drag and drag coefficient from those with the original bill. **A** △*D* (%); **B** *C_D_*. For the sailfish, ⋄, original bill (shown only in **B**); ○, short bill; □, long bill; △, smooth bill; ▽, rough bill. For the swordfish, ⋄, original bill (shown only in **B**); •, short bill. Here, we use the Reynolds number based on the body length, *Re_BL_*, because of the change in the bill length. Error bars are also plotted in this figure.

As mentioned in the **Introduction**, possibilities of reducing drag by the bill have been suggested before [5], [7], [10], [29], [30], [38]. To investigate the effect of bill on the drag, the drags on the sailfish and swordfish are measured by modifying the shapes of bills. Again, the fishes without the median and paired fins are considered to isolate the effect of the bill on the drag. Figure 7A shows the variation of the drag in percentage from that with the original bill, where the bills are modified in length or in roughness. As mentioned earlier (see *Taxidermy specimens*), four artificial bills (shorter, longer, smoother and rougher ones) are considered for the sailfish, and only a shorter one is tested for the swordfish. As shown, with shorter or smoother bill, the drag on the sailfish becomes smaller than that with the original one, whereas it becomes larger with longer or rougher one. However, the amounts of drag variation are quite small (within ±2%), which is within the experimental uncertainty. Likewise, the drag on the swordfish with shorter bill is smaller by 1∼2.5% than that with the original one, and the difference between them is clearer owing to the long original bill of the swordfish. When the drag coefficient is computed based on the wetted area including that of bill, however, the swordfish with the original bill has lower drag coefficient by 4∼5% than that with shorter bill, whereas the shape of bill does not make any difference in the drag coefficient for the sailfish (Figure 7B).

**Figure 8:**
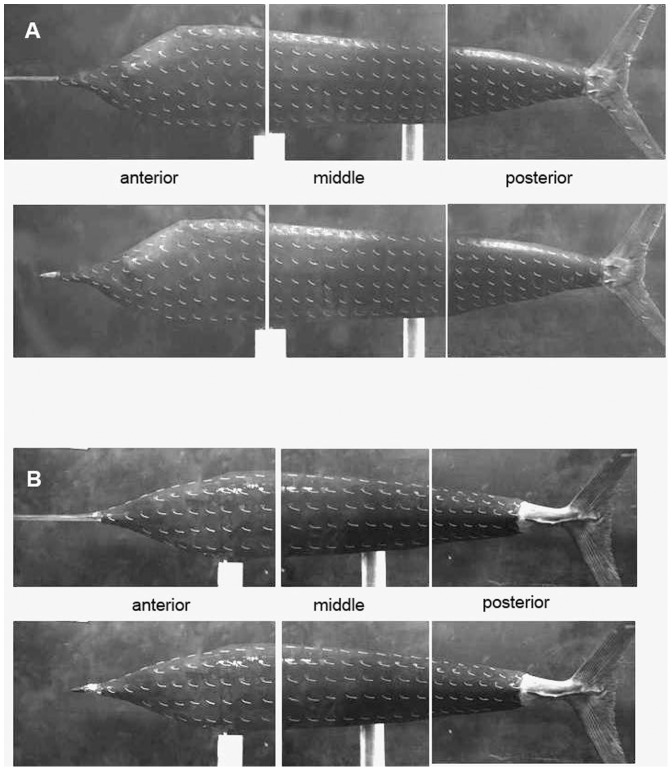
Tuft visualizations on the body surface. In both **A** sailfish and **B** swordfish, the upper and lower figures correspond to the fish with the original and shorter bills, respectively. Here, the body of each fish is wrapped by a thin tape and then coated in black paint for better visualization.

Figures 8A and B show the surface-flow patterns on the bodies of the sailfish and swordfish, respectively, obtained from tuft visualizations. We perform tuft visualizations for all the cases considered in the drag measurement, but there is essentially no difference in the flow patterns from those shown in Figure 8. All the threads over the entire surface of each fish do not show any indication of flow separation, confirming the result from the velocity measurements shown in Figures 5 and 6. Flow separation does not occur even with shorter bill at the cruising condition, so there is no reason to increase turbulence using the bill for reducing the form drag. This result is contrary to the previous conjectures [5], [29]. Aleyev [7] also performed tuft visualization on the models of sailfish and swordfish, and showed that flow separation does not occur from the body. However, it should be noted that the present result is valid only at the cruise speed. The role of the bill at the maximum speed is still to investigate, which is not possible with the present experimental setup.

**Figure 9:**
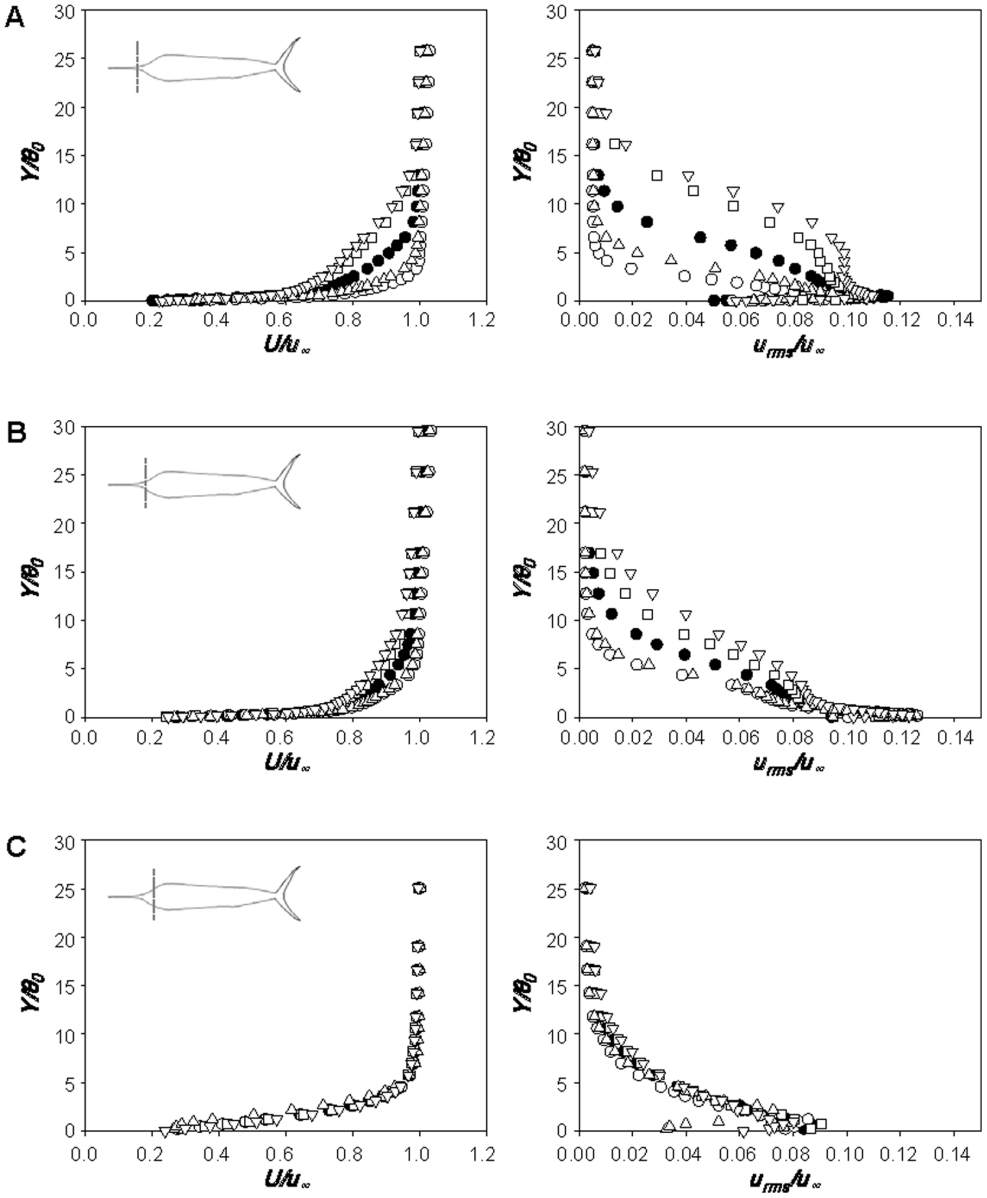
Velocity profiles over the body surface of the sailfish with different bills. Profiles of the mean streamwise velocities (left) and rms streamwise velocity fluctuations (right) along the body of sailfish measured at *X_1_/BL*  =  **A** 0.029 (5 cm), **B** 0.086 (15 cm) and **C** 0.144 (25 cm): •, original bill; ○, short bill; □, long bill; △, smooth bill; ▽, rough bill. Here, *X_1_* is the streamwise distance from the tip of lower jaw, *Y* is the wall-normal distance from the surface and *θ_0_* is the momentum thickness with the original bill. The measurement locations are also plotted in each figure. The velocities are measured along the centerline of dorsal part at *Re_BL_*  = 2.32×10^6^.

Figure 9 shows the profiles of mean streamwise velocity (left) and rms streamwise velocity fluctuations (right) at *X_1_/BL*  = 0.029, 0.086 and 0.144 along the centerline of dorsal part of the sailfish with original, shorter, longer, smoother and rougher bills, respectively, at *Re_BL_*  = 2.32×10^6^. Here, *X_1_* is the streamwise distance from the tip of lower jaw. All these streamwise locations are ahead of the point of maximum body thickness. As shown in Figure 9A, the boundary layer flow is not laminar even at *X_1_/BL*  = 0.029 and is much closer to turbulent flow, irrespective of the bill shape. It is known that the critical Reynolds number for laminar-to-turbulent transition on a smooth flat plate is 3.5×10^5^ to 10^6^ and distributed roughness causes transition to turbulent boundary layer at *u_∞_k*/*v*>120 [62], where *k* is the roughness height. The average size of roughness on the bill is larger (∼667*v*/*u_∞_* at *u_∞_* = 20 m/s) than this critical value, and thus turbulent boundary layer flow can be observed even at *X_1_/BL*  = 0.029, although its corresponding local Reynolds number for the shorter bill (*Re_X_*; *X* is the distance from the tip of bill) is only about 1.20×10^5^. On the other hand, the smoother bill having no roughness is long enough for the transition to turbulence (*Re_X_*  = 4.53×10^5^ at *X_1_/BL*  = 0.029), and the concave geometry of the head part or disturbances from protrusions existing around the mouth may also cause the boundary layer to grow to be turbulent. Although the boundary layer flows are turbulent at this location for all the bills considered, there exist clear differences in the profiles of mean and rms velocities among different bill shapes (Figure 9). The momentum thickness and turbulence intensity at *X_1_/BL*  = 0.029 are larger when the bill is longer or rougher (Figure 9A). However, shortly after this location, the differences become smaller (Figures 9B and C) and the mean and rms velocities are nearly identical irrespective of the bill shape (Figure 9C). This means that the flow development along the whole fish body is mainly determined by the shape of body including the head part, but not much by the bill itself. Another interesting observation from Figure 9C is that the mean velocity profile becomes flattened and rms velocity fluctuations are greatly reduced at *X_1_/BL*  = 0.144 owing to the favorable pressure gradient formed there and before. Therefore, it is quite clear that the bill produces a turbulent boundary layer flow earlier as conjectured by Ovchinnikov [5] and Webb [29], but these flow characteristics do not persist farther downstream owing to the favorable pressure gradient formed by the head shape of the fish.

**Figure 10:**
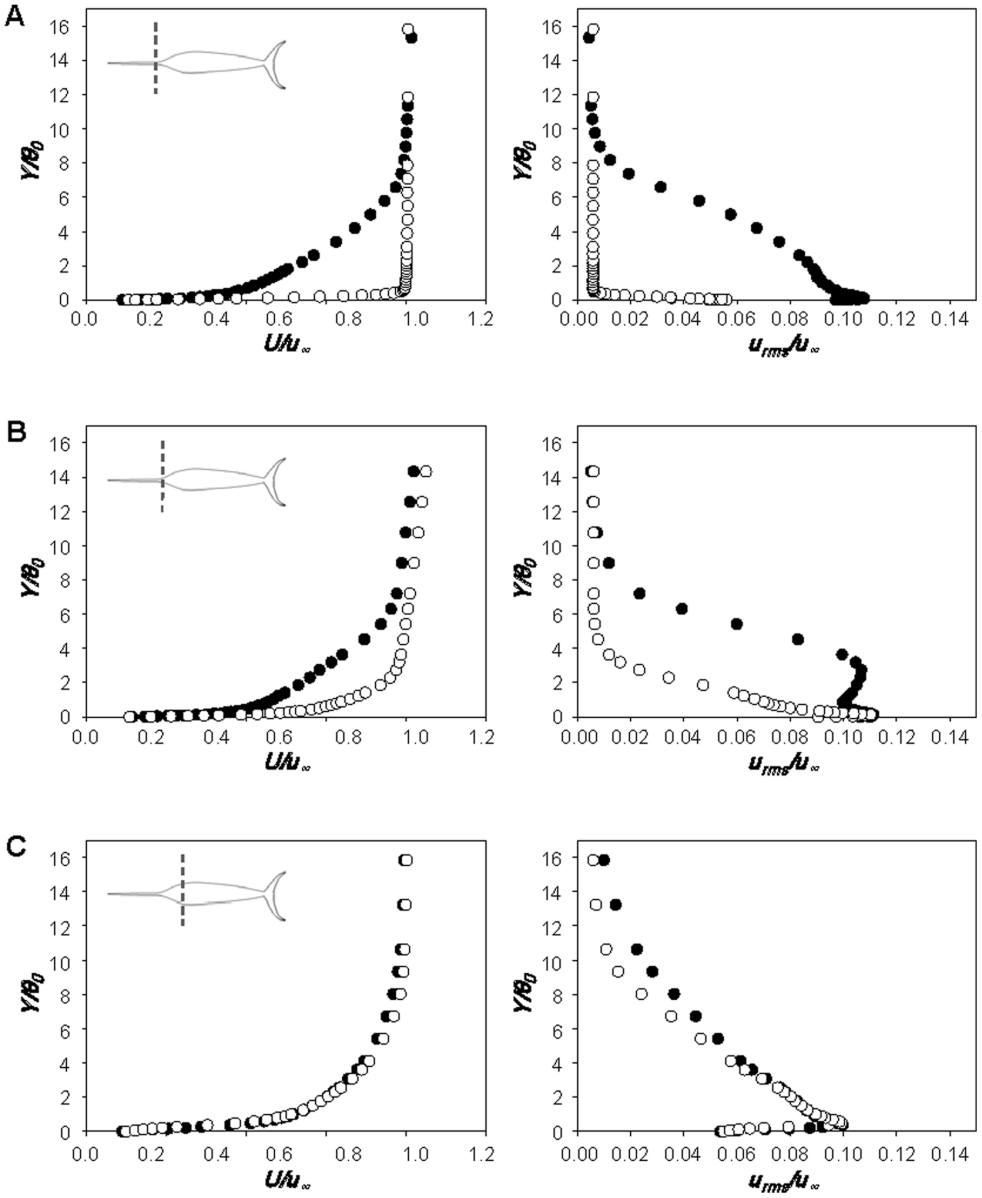
Velocity profiles over the body surface of the swordfish with different bills. Profiles of the mean streamwise velocities (left) and rms streamwise velocity fluctuations (right) along the body of swordfish measured at *X_1_/BL*  =  **A** -0.023 (-3 cm), **B** 0.039 (5 cm) and **C** 0.219 (28 cm): •, original bill; ○, short bill. Here, *X_1_* is the streamwise distance from the tip of lower jaw, *Y* is the wall-normal distance from the surface and *θ_0_* is the momentum thickness with the original bill. The measurement locations are also plotted in each figure. The velocities are measured along the centerline of dorsal part at *Re_BL_*  = 1.71×10^6^.

Figure 10 shows the profiles of mean streamwise velocity (left) and rms streamwise velocity fluctuations (right) along the centerline of dorsal part of the swordfish with the original and shorter bills, respectively, at *Re_BL_*  = 1.71×10^6^. At *X_1_/BL*  = −0.023 (Figure 10A), the boundary layer profile with the original bill (*Re_X_*  = 7.07×10^5^) is close to that of a turbulent boundary layer, showing that the bill indeed produces a turbulent flow at an early streamwise location. However, with the shorter bill, the boundary layer flow at this location (*Re_X_*  = 3.33×10^4^) is close to laminar or transient flow. At this location, the shape factors of boundary layer velocity profiles are about 1.51 and 1.95 with the original and shorter bills, respectively (1.3 and 2.6 for turbulent and laminar boundary layer flows over a flat plate with zero pressure gradient [45]). At *X_1_/BL*  = 0.039 (Figure 10B), the boundary layer grows and turbulence intensity increases. At *X_1_/BL*  = 0.219 (Figure 10C), the flows with original and shorter bills are subject to a strong favorable pressure gradient owing to the shape of head part, and their flow characteristics become nearly the same irrespective of the bill shape, reaching the same conclusion obtained from the sailfish. From the present velocity measurement, it is concluded that the bill has a role in reducing the skin friction in the anterior part of fish, but the skin-friction reduction by the bill does not occur over the entire body. Therefore, the overall drag is nearly unchanged in the presence of the bill because the area in which the skin friction is reduced is small and the bill itself generates additional drag.

### Concluding remarks

Motivated by their fast swimming speeds and peculiar shapes, we investigated the hydrodynamic characteristics of the sailfish and swordfish at their cruise speeds by installing taxidermy specimens in a wind tunnel, directly measuring the drags on the bodies, and probing the boundary layer velocities above the body surfaces. The drag coefficients of the sailfish and swordfish at the cruise conditions were about 0.0075 and 0.0091 based on the free-stream velocity and wetted area, respectively. These values of the drag coefficient were smaller than those of dogfish and small-size trout and comparable to those of tuna and pike. The median and paired fins have been known as effective devices for enhancing the maneuverability or stability of the fish, but they inevitably increase the drag on the fish. Thus, the sailfish usually folds down the first dorsal, first anal, and pelvic fins in cruising or gliding. However, it is still unknown why the swordfish have not developed to depress those fins unlike the sailfish. We also found that the boundary layer flow characteristics of both fishes are quite similar to each other: i.e., turbulent boundary layer flows exist over most of body surfaces even at their cruise conditions and flow separation does not occur on the whole body surfaces.

The sailfish and swordfish have distinct morphological features from those of other fast fishes. For the sailfish, many V-shaped protrusions were found on the body skin and were tested for possible skin-friction reduction, resulting in nearly no drag reduction by the protrusions [31]. In the present study, we examined another possible role of the V-shaped protrusions in delaying flow separation (if any) by performing tuft visualizations and measuring the drag forces on the body with and without the protrusions, respectively. Even in the absence of the protrusions, flow separation did not occur from the whole body surface, and the drag on the sailfish without the protrusions was even slightly smaller than that in their presence. This result indicates that the V-shaped protrusions on the sailfish skin do not make any role in reducing the drag at the cruise condition.

Another interesting morphological feature of the sailfish and swordfish is the bill. The roles of bill have been conjectured as a drag-reduction device by delaying the flow separation [5], [29] or reducing the skin friction on the main body [7], [30], [38]. In the present study, we found that the drags with and without the bill are nearly the same. The bill generated a turbulent boundary layer flow at the initial part of head and reduced the skin friction only at the anterior part. However, this effect of skin-friction reduction did not persist farther downstream, because the strong favorable pressure gradient after mid-head part significantly changed the boundary layer characteristics and the boundary layer velocity profiles with and without the bill were nearly the same at the end of head part of each fish. Furthermore, the bill itself generated additional drag which may be compensated with the reduction of skin friction at the anterior part, resulting in nearly no change in the overall drag at the cruise speed. Nevertheless, it was interesting to note that the drag coefficient based on the wetted area is lower with original bill of swordfish than that with shorter one, whereas the drag coefficients of the sailfish were nearly insensitive to the change in the bill shape.

Lastly, it should be mentioned that the present conclusions were obtained at the conditions of cruise speeds in gliding postures. The hydrodynamic characteristics of the sailfish and swordfish at the maximum speed or during undulatory swimming motion are important subjects to pursue in the near future, which we could not study owing to the technical difficulty of achieving high speed from our experimental setup or lack of information on their swimming kinematics, respectively.
